# Imaging of carotid artery vessel wall edema using T2-weighted cardiovascular magnetic resonance

**DOI:** 10.1186/1532-429X-16-22

**Published:** 2014-03-04

**Authors:** Lars Ølgaard Bloch, Anne Yoon Krogh Grøndal Hansen, Steen Fjord Pedersen, Jesper Langhoff Honge, Won Yong Kim, Esben Søvsø Szocska Hansen

**Affiliations:** 1 Department of Cardiology, Aarhus University Hospital Skejby, Brendstrupgaardsvej 100, DK-8200 Aarhus N, Denmark; 2 MR Research Centre, Aarhus University Hospital Skejby, Brendstrupgaardsvej 100, DK-8200 Aarhus N, Denmark; 3 Department of Cardiothoracic & Vascular Surgery, Aarhus University Hospital Skejby, Brendstrupgaardsvej 100, DK-8200 Aarhus N, Denmark

**Keywords:** Cardiovascular magnetic resonance, Atherosclerosis, Inflammation, Carotid artery

## Abstract

**Background:**

Atherothrombosis remains a major health problem in the western world, and carotid atherosclerosis is an important contributor to embolic ischemic strokes. It remains a clinical challenge to identify rupture-prone atherosclerotic plaques before clinical events occur. Inflammation, endothelial injury and angiogenesis are features of vulnerable plaques and may all be associated with plaque edema. Therefore, vessel wall edema, which can be detected by 2D T2-weighted cardiovascular magnetic resonance (CMR), may be used as a dynamic marker of disease activity in the atherosclerotic plaque. However, 2D imaging is limited by low spatial resolution in the slice-select direction compared to 3D imaging techniques. We sought to investigate the ability of novel 3D techniques to detect edema induced in porcine carotid arteries by acute balloon injury compared to conventional 2D T2-weighted black-blood CMR.

**Methods:**

Edema was induced unilaterally by balloon overstretch injury in the carotid artery of nine pigs. Between one to seven hours (average four hours) post injury, CMR was performed using 2D T2-weighted short-tau inversion recovery (T2-STIR), 3D volumetric isotropic turbo spin echo acquisition (VISTA) and 3D T2 prepared gradient-echo (T2prep-GE). The CMR images were compared in terms of signal-to-noise ratio (SNR) and contrast-to-noise (CNR) ratio. Furthermore, the presence of vessel wall injury was validated macroscopically by means of Evans Blue dye that only enters the injured vessel wall.

**Results:**

All three imaging sequences classified the carotid arteries correctly compared to Evans Blue and all sequences demonstrated a significant increase in SNR of the injured compared to the non-injured carotid vessel wall (T2-STIR, p = 0.002; VISTA, p = 0.004; and T2prep-GE, p = 0.003). There was no significant difference between sequences regarding SNR and CNR.

**Conclusion:**

The novel 3D imaging sequences VISTA and T2prep-GE perform comparably to conventional 2D T2-STIR in terms of detecting vessel wall edema. The improved spatial coverage of these 3D sequences may facilitate visualization of vessel wall edema to enable detection and monitoring of vulnerable carotid atherosclerotic plaques.

## Background

Stroke is a leading cause of disability and the fourth leading cause of death in the US [1]. About 87% of all strokes are ischemic [1], of which carotid atherosclerotic lesions are considered to account for about 25% [2]. Inflammation plays a key role in all stages of atherosclerosis and subsequently may lead to atherothrombosis [3,4]. Accordingly, on-going research is aimed to find imaging methods that can identify the inflammatory status of the plaque. Several imaging modalities have been used in this respect. Positron emission tomography with detection of arterial fluorodeoxyglucose uptake has been shown to correlate with the amount of macrophages [5-7] in the atherosclerotic plaque providing a measure of the inflammatory burden. Also, carotid inflammation can be detected by contrast-enhanced cardiovascular magnetic resonance using targeted gadolinium agents [8,9] or ultra-small particles of iron oxide [10,11]. Tearney et al. showed that the optical coherence tomography signal correlates with the amount of macrophages in the plaque [12]. Other invasive methods that may be able to identify inflammation in atherosclerotic lesions include near-infrared spectroscopy [13] and thermography [14,15]. However, it would be preferable to have a non-contrast dependent non-invasive imaging method that may identify the inflammatory status of the plaque to monitor plaque development and response to therapy.

Some of the characteristic features of vulnerable plaques include inflammation, endothelial injury and neoangiogenesis [3,4,16,17] which may all be associated with vessel wall edema. Previous studies have demonstrated that vessel wall edema in the carotid and coronary arteries can be detected by 2D T2-weighted (T2W) short-tau inversion recovery (T2-STIR) [18,19]. Furthermore, evidence now exists that vessel wall edema is present and can be detected by 2D T2-STIR within the culprit lesion of the coronary arteries in patients with acute coronary syndrome [20,21]. Since 2D sequences are limited by poor spatial resolution and reduced coverage, we sought to determine the ability of 3D T2W black-blood MRI sequences to detect edema induced in porcine carotid arteries by acute balloon injury [22] compared with conventional 2D T2-STIR. Zhang et al. demonstrated in human femoral arteries that 3D turbo spin echo (TSE) with variable flip angles enabled superior vessel wall visualization, superior blood signal suppression, and comparable vessel wall morphological measurements compared to 2D TSE T2W imaging [23]. Thus, we included a Volumetric ISotropic Turbo spin echo Acquisition (VISTA) in the imaging protocol of our study. We also included a 3D T2 prepared gradient-echo (T2prep-GE) sequence with motion sensitized gradients to ensure black-blood properties.

## Methods

### Animal model

Nine female Danish Land Race pigs weighing about 40 kg were used for the experiments. The pigs were treated in accordance with the Danish Law on Animal Experiments. The pigs were pre-sedated with an intramuscular injection of azaperone, (4 mg/kg) and midazolam (0,5 mg/kg) which allowed intravenous access via an ear vein. Midazolam (0,5 mg/kg) and ketamine (5 mg/kg) were administered intravenously before endotracheal intubation and coupling to a ventilator (tidal volume 450 mL, respiratory rate 12/min). Anaesthesia was maintained with infusion of propofol (3 mg/kg/h) and fentanyl (15–25 μg/kg/h). By a surgical cut down in the groin, the right common femoral artery was exposed and a 10 F introducer sheath was inserted into the artery. Then a bolus injection of heparin (100 IU/kg) was given through the sheath. For the balloon overstretch injury, a 12 mm over-the-wire angioplasty balloon was randomized to be placed in either the left or right carotid artery. The injury was induced by inflation to 5–8 atmospheres for a few seconds, which was empirically chosen to create vessel wall edema while minimizing the risk of dissection and haemorrhage. The inflated balloon was pulled gently back and forth a few millimetres before deflation to denudate the endothelium. Table 1 summarizes the experimental protocol.

**Table 1:** Overview of experimental protocol

**Pig no.**	**Carotid artery randomized for balloon injury**	**Balloon size (diameter in mm)**	**Applied pressure (atm)**	**Hours after balloon injury when MR scanning was performed**	**Minutes of Evans Blue circulation after 45 minutes of infusion**
1	Right	12	8	5	60
2	Right	10	12	6	90
3	Left	12	7	7	90
5	Left	12	6	2	90
6	Left	12	6	6	90
7	Right	12	6	1	90
8	Right	12	7	1	90
9	Right	12	6	3,5	90

Pig no. 4 developed dissection and occlusion of the right carotid artery, so the MR images of this pig were withdrawn from further data analysis. Thus, eight pigs were included in the analysis. To compensate for the slightly smaller balloon (10 mm) used for pig no. 2, a higher pressure was applied to get the same amount of dilation as estimated on x-ray angiography.

### Magnetic resonance imaging

The pigs were kept in general anaesthesia and one to seven hours (average four hours) after the balloon injury, the carotid CMR protocol was performed on a 1.5 T system (Achieva, Philips Healthcare, Best, The Netherlands) using a five channel sense cardiac coil. All the pigs were imaged in the supine position. A standardized imaging protocol was applied. After an initial localizer scan, an axial 2D time-of-flight CMR angiography was conducted to identify the carotid arteries. Then the three black-blood imaging sequences were all acquired in the axial plane according to scanning parameters summarized in Table 2. The field of view (FOV) covered the carotid arteries from the common carotid trunk to a couple of centimeters above the carotid bifurcation. The edema scanning was performed using 2D T2-weighted short-tau inversion recovery (T2-STIR) [24], 3D volumetric isotropic turbo spin echo acquisition (VISTA) [23] and 3D T2 prepared gradient-echo (T2prep-GE) [25] with motion sensitized gradients to ensure black-blood properties [26] and multi-echo 2 point DIXON for fat-water separation less sensitive to B0 inhomogeneities compared to spectral selective saturation prepulses [27]. The Motion-Sensitized Driven-Equilibrium (MSDE) Velocity Encoding (VENC) value was set to 2.1 cm/s in the slice-select direction. For the VISTA sequence, the variable-flip-angle refocusing pulse train was used, with α_min_ of 18°and α_max_ of 120°. Cardiac triggering was used for the T2-STIR and T2prep-GE sequences to obtain images in the mid-diastolic phase. Constant level appearance (CLEAR) was used for the data acquisition of all three imaging sequences. Since CLEAR alters the noise appearance by creating a constant intensity level on the entire image, SNR should be considered an estimate. However, over- or underestimation of the SNR is likely to be within the same range for all three sequences and therefore it should not affect the statistical comparisons.

**Table 2:** Imaging parameters for T2-STIR, VISTA, and T2prep-GE sequences

**MRI sequence**	**T2-STIR**	**VISTA**	**T2prep-GE**
**Technique**	**2D black blood, TSE**	**3D black blood, TSE**	**3D black blood, GE**
TE, ms	100	366	1.88/3.5
TR, ms	1800	1400	5.9 ms
Echo train length	30	177	-
Matrix size	200 × 200	200 × 200	200 × 200
Number of slices	50	397	396
Spatial resolution, mm^3^	1.5 × 1.57 ×4	1 × 1 × 1	1 × 1 × 1
Slice gap, mm	4	-	-
Number of signal average (NSA)	2	4	2
Flip angle	90	-	15
TFE factor	-	-	16
Scan time, minutes	24	16	17

T2-STIR; T2-weighted Short Tau Inversion Recovery. VISTA; Volumetric ISotropic Turbo spin echo Acquisition. T2prep-GE; T2 prepared gradient echo. TSE; Turbo Spin Echo. TFE; Turbo Field Echo.

### Evans blue dye

Immediately after completion of the CMR examination, the pigs were taken back to the surgical facility. Evans Blue dye (EBD) was used to validate the CMR findings of vessel wall edema by identifying the location of the balloon-injured segment of the artery ex vivo and verifying macroscopically that the balloon injury had indeed increased the permeability of the artery. After intravenous injection, the dye binds to albumin. Only in case of a defect endothelial barrier, albumin with attached EBD will enter the vessel wall and the surrounding tissue. Injury to the vessel wall, however, increases the vessel wall permeability and allows albumin to leak out of the vessel together with edema-forming fluid. The EBD solution was made by dissolving 2 g of EBD in 50 mL of saline. EBD was administered intravenously during 45 min. and then circulated in the bloodstream for additional 90 min. In pig no.1, EBD only circulated for 60 min., which was increased to 90 min. for the remaining pigs to increase the wash out of EBD. Afterwards the pigs were euthanized by an overdose of pentobarbital and the carotid arteries were harvested. The carotid arteries were fixated in formaldehyde and kept in phosphate buffer for a few days until further examination. To register any possible uptake of EBD, the carotid arteries were cut open longitudinally and photographed with a digital camera (Nikon, Tokyo, Japan).

### Data analysis

#### Cardiovascular magnetic resonance

The luminal and outer arterial wall boundary were delineated manually by drawing regions of interest (ROIs) on the T2-STIR, VISTA and T2prep-GE images using Osirix version 5.0.2. The vessel wall was determined by subtracting the inner luminal ROI from the outer ROI. The segmented area covered the injured and non-injured carotid arteries from the common carotid trunk to the carotid bifurcation. The CMR images were graded as 0 (non-diagnostic), 1 (poor), 2 (fair), 3 (good) or 4 (excellent) according to the delineation of the vessel wall and overall image quality. Images graded as 0 or 1 were discarded from further analysis and typically included images in the outer parts of the FOV at the borders of the coil range. For the T2-STIR sequence, 98.6% (281/285) of the segmented slices were graded ≥ 2, for the VISTA sequence it was 97.2% (1976/2032) and for T2prep-GE it was 92.2% (1823/1977) of the segmented slices. A reference ROI was placed in a homogenous region of corresponding muscles on the two sides, mostly the sternocleidomastoid muscle. However, this muscle was not present in images in the outer parts of the FOV, and in this case corresponding muscles of the lateral or posterior neck was used. A reference ROI was also placed in the image background on both sides. The normalized signal intensity (SI_mean_) of the vessel wall on each side was calculated as SI_vessel wall_/SI_muscle_ for each slice and an average value was calculated for each carotid vessel. Contrast to noise ratio (CNR) was calculated for the T2-STIR, VISTA and T2prep-GE images according to the signal to noise ratios (SNR) of injured and non-injured carotid artery:

$$\begin{array}{l} \text{CNR}={\mathrm{SNR}}_{\text{injured}}–{\mathrm{SNR}}_{\text{non}-\text{injured}},\\ \mathrm{SNR}={\text{SI}}_{\text{mean}}/{\text{SD}}_{\text{noise}} \end{array}$$

Thus, the SNR was calculated as the ratio between signal intensity of the vessel wall and signal intensity of muscle times the SD_noise_ to ensure independence of different anatomical positions relative to the surface coil. All slices were segmented by one observer (LØB) who was blinded to the injury status. To test the influence of variation in vessel wall segmentation, all outer ROI’s were increased by 10% in all slices since this was often the most difficult ROI to place correctly due to adjacent anatomical structures such as the thyroid gland and lymph nodes with iso-intense SI or edema leaking out of the vessel into the surroundings, cf. Table 3. An in-house MATLAB R2012b script was developed to extract ROI data and to perform statistical analysis. An additional script used ROI data to produce in-plane images of the carotid arteries.

**Table 3:** Results for enlarged ROIs to test for the importance of the accuracy of segmentation

**Imaging sequence**	**Paired t-test p-value**	**CI**_**95**_	**SNR**_**injured**_	**SNR**_**non-injured**_	**CNR**
T2STIR	0.005	8.05-29.9	23.0	17.9	5.1
VISTA	0.009	5.5-27.7	24.3	19.0	5.4
T2prep-GE	0.006	4.4-18.0	21.5	17.3	4.2

#### Evans blue dye

The digital photos of the carotid arteries were reviewed by two observers (LØB, ESSH) for uptake of EBD. The relative distances between anatomical landmarks were found by segmentation of the photos in Osirix version 5.0.2.

The CMR images of the injured and non-injured carotid arteries were matched to the macroscopic staining by EBD of the carotid arteries for each of the three CMR sequences using anatomical landmarks, such as the distance from the common carotid trunk and the upper carotid bifurcation as well as gross morphological features of the lumen and vessel wall.

### Statistical analysis

Each pig was considered a paired set of data consisting of an injured and non-injured carotid artery. Student’s paired t-test was used to compare SNR between injured and non-injured vessel. SNR and CNR for all three imaging sequences are presented as mean + 95% confidence intervals (CI_95_). We compared differences in SNR and CNR between the sequences using one-way analysis of variance (ANOVA). All calculations were made using MATLAB R2012b Statistics Toolbox.

## Results

**Figure 1:**
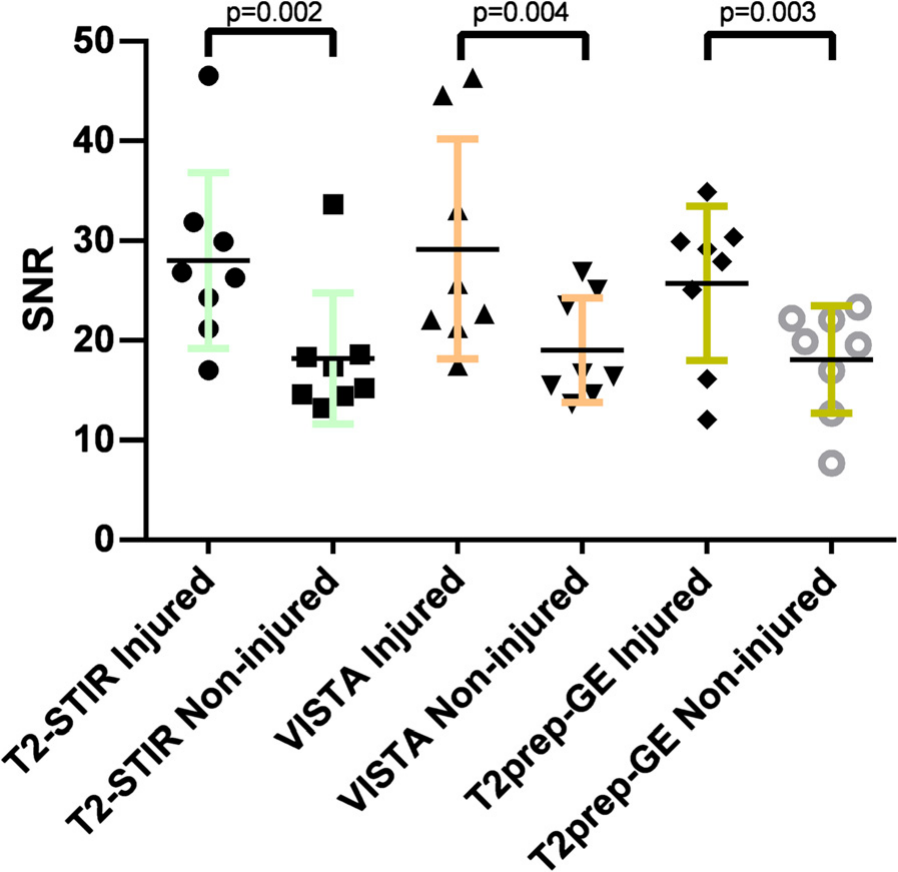
**Signal-to-noise ratio (SNR) of the injured and non-injured carotid artery vessel wall.** The SNR of the injured versus non-injured carotid artery vessel wall of the eight pigs using T2-STIR, VISTA and T2prep-GE sequences are shown. The horizontal line represents the median value. The p-value refers to the paired t-test comparing SNR for injured vs. non-injured carotid artery.

**Figure 2:**
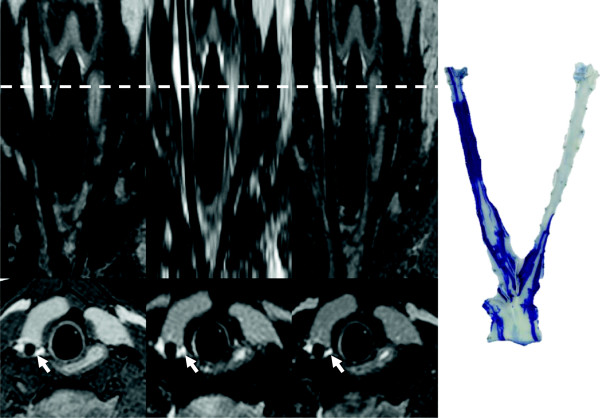
**Cardiovascular magnetic resonance images and Evans Blue image of pig no. 8.** The top panel shows reformatted in-plane images of the carotid arteries from pig no. 8. The T2-STIR, VISTA and T2prep-GE images are shown from left to right. The bottom panel displays the corresponding cross-sectional images. The right side of the figure shows ex vivo macroscopic visualization of the carotid arteries showing the vessel wall injury colored by EBD. The balloon was inflated in the right carotid artery but the tip of the catheter also induced injury within the vessel wall of the proximal part of the left carotid artery.

All CMR scans were performed successfully. All three imaging sequences classified the injured and non-injured carotid artery correctly according to the EBD staining of the carotid arteries and demonstrated a significant increase in the SNR of the vessel wall in the injured compared to the non-injured carotid artery (Figure 1). The results of the paired t-tests and SNR and CNR for all three imaging sequences are presented in Table 4. Typical images of the carotid arteries showing STIR, VISTA and T2prep-GE images together with the corresponding EBD image are shown in Figure 2.

**Table 4:** SNR and CNR values for T2-STIR, VISTA and T2prep-GE

	**Paired t-test p-value**	**Mean difference**	**CI**_**95**_	**SNR**_**injured**_	**SNR**_**non-injured**_	**CNR**
T2-STIR	0.002	9.8	[6.9-12,8]	22.5, [16.8-28.2]	17.3, [12.4-22.3]	5.15, [3.0-7.3]
VISTA	0.004	6.9	[4.4-15.9]	23.8, [17.1-30.6]	18.3, [14.2-22.3]	5.6, [1.7-9.5]
T2prep-GE	0.003	4.6	[3.8-11.5]	20.9, [17.0-24.9]	16.7, [12.8-20.5]	4.3, [1.9-6.7]

The p-value refers to the paired t-test comparing SNR for injured vs. non-injured carotid artery.

ANOVA showed no significant differences in SNR at the p < 0.05 level for the three sequences neither for the injured carotid artery [F(2,21) = 0.38, p = 0.6917] nor for the non-injured carotid artery [F(2,21) = 0.19, p = 0.8315]. ANOVA for CNR between the three sequences showed no significant differences [F(2,21) = 0.3, p = 0.7447]. Enlargement by 10% of all ROIs delineating the outer carotid wall did not change the significance of the results as is seen from Table 3.

## Discussion

In this study, we have demonstrated the ability of the 3D CMR sequences VISTA and T2prep-GE to visualize vessel wall edema in porcine carotid arteries following acute balloon injury. A significant increase in SI within the injured compared to the non-injured carotid artery vessel wall was observed, which was comparable to the conventional 2D T2-STIR imaging sequence. Due to the inherent higher SNR, the 3D sequences allowed for 1 mm^3^ isotropic spatial resolution with a shorter scan time compared to 2D T2-STIR.

To the best of our knowledge, this is the first systematic comparison of 2D and 3D imaging sequences for the visualization of vessel wall edema with implications for further clinical studies to unravel the importance of plaque edema in the natural history of vulnerable atherosclerotic phenotype.

Pedersen et al. have shown that gadofosveset, an albumin-binding gadolinium contrast agent, detects endothelial damage and angiogenesis in this experimental model of vessel wall edema [28]. Phinikaridou et al. demonstrated the presence of gadofosveset in the vessel wall of a murine atherosclerosis model [29]. Uptake of gadofosveset correlated with EBD staining, morphological changes of endothelial cells, and widening of cell-cell junctions measured by electron microscopy suggesting an increased permeability [29]. Also, Pedersen et al. used fibrinogen as a surrogate marker of edema with the same rationale as described for albumin and demonstrated a linear correlation between the area of fibrinogen staining on immunohistochemistry and the relative SI on CMR (ρ = 0.93, P < 0.001) and also demonstrated agreement between hyperintense SI on T2-STIR images and uptake of EBD (Χ^2^ = 17.14, P < 0.001) [19]. Thus, balloon overstretch injury is a validated experimental method causing vessel wall edema. Since it is difficult to histologically verify edema, EBD is used experimentally to assess vessel wall permeability and edema [18,19,29]. In this study, we detected the macroscopic presence of EBD in the vessel wall to locate the balloon injury and used this as reference in the comparison of the CMR sequences. The mechanical balloon injury to the vessel wall was twofold as both overstretching of the vessel wall and pulling the deflated balloon backwards will inevitably cause injury to the vessel wall. Consequently, all carotid segments exposed to the balloon or just the catheter itself demonstrated uptake of EBD. This is demonstrated in the EBD image for pig no. 8 in Figure 2 where the tip of catheter had also been placed in the proximal part of the non-injured artery. Interestingly, it seems that endothelial denudation is enough to locally increase vessel wall permeability, as has been suggested in previous studies [28,29].

It has been shown that plaque rupture can occur also in low-grade stenosis and that the degree of luminal stenosis is not an optimal predictor of future events [30]. In carotid atherosclerosis, significant luminal stenosis may predict one out of four strokes in symptomatic patients [31] but only one out of ten strokes in asymptomatic patients [32]. Angiography methods may underestimate the atherosclerotic burden due to expansive arterial remodelling [33] and by measuring the luminal stenosis relative to the supposedly normal adjacent sites [30]. Nevertheless, luminal carotid artery stenosis remains the primary criteria for surgical therapy in symptomatic patients. CMR is emerging as a comprehensive non-invasive imaging modality for the assessment of the carotid and coronary vessel wall [34-37]. Multicontrast carotid CMR is a histologically validated method for morphological plaque classification [35,36]. However, due to the complexity of the scanning protocols and the carotid plaque analysis, more widespread use has been limited. Our approach using heavily T2-weighted sequences to visualize plaque edema may provide a more simplified measure of plaque vulnerability. Further studies in patients with carotid atherosclerosis are needed to verify the possible clinical impact of visualizing carotid plaque edema. Improvements have been made with the use of gadolinium contrast agents detecting several features of the vulnerable plaque [38-40] as well as the use of targeted contrast agents in molecular imaging [8,9]. However, a non-contrast dependent method would eliminate potential contrast-related complications. CMR may also be used in the estimation of the biomechanical stresses within the plaque [41,42] which may cause mechanical instability and thereby possibly influence plaque vulnerability. Thus, different mechanisms may act in concert and different imaging approaches are needed for a comprehensive evaluation of plaque vulnerability.

Our group has shown that carotid and coronary vessel wall edema can be detected using 2D T2-STIR [18,19]. However, clinical application using 2D TSE sequences may be hampered by the relatively low spatial resolution in the slice-select direction making 2D images more prone to partial volume effects and the need for a relatively long acquisition time to achieve sufficient SNR and/or in-plane spatial resolution [23]. In this regard, 3D imaging protocols such as the proposed VISTA and T2prep-GE sequences have some inherent advantages with intrinsic high SNR that can be utilized to yield isotropic spatial resolution and improved anatomic coverage of plaque components. Furthermore, imaging 3D isotropic volumes, multi-planar reformation allows for reformatting images in arbitrary planes, which is often helpful for depicting anatomical structures [23]. Zhang et al. reported that VISTA and 2D TSE performed similarly in measurements of wall and lumen volume and that VISTA demonstrated significantly higher CNR and markedly reduced scan time [23]. The T2prep-GE technique has been applied for cardiac magnetic resonance [43,44] and the fat-water separation obtained with this sequence may also be useful in atherosclerosis imaging.

### Limitations

In terms of applying these results to atherosclerosis imaging, it may be considered a limitation to study vessel wall edema in a non-atherosclerotic animal model of balloon injury. However, for validating CMR sequences, it may be considered advantageous to be able to visualize vessel wall edema in isolation. The balloon injury may cause more edema than there will be present in an atherosclerotic plaque but even very subtle injury to the vessel caused by pulling back the deflated balloon was reliably detected by CMR in accordance with uptake of EBD. Since both the thyroid gland and lymph nodes around the proximal part of the carotid arteries would appear bright on the T2W images, accurate segmentation of the carotid vessel wall was sometimes difficult. However, as seen from Table 3 even deliberately enlarging the outer manual delineation of the vessel wall did not change the significance of the results illustrating the robustness of the segmentation and comparison method of injured versus non-injured vessel.

## Conclusion

We have demonstrated the ability of 3D T2W black-blood imaging sequences to detect vessel wall edema in porcine carotid arteries following acute balloon injury. Clinical application of vessel wall edema visualization in carotid atherosclerosis may benefit from 3D imaging sequences compared to conventional 2D imaging techniques due to inherently higher SNR allowing for isotropic spatial resolution. Plaque edema in atherosclerosis may prove to be a useful surrogate marker in the identification of the vulnerable plaque, and further studies are needed to determine the clinical applicability of vessel wall edema.

## Abbreviations

2D: Two-dimensional; 3D: Three-dimensional; α: Flip angle; CLEAR: Constant level appearance; CMR: Cardiovascular magnetic resonance; CNR: Contrast-to-noise-ratio; EBD: Evans blue dye; FOV: Field of view; MSDE: Motion-sensitized driven-equilibrium; T2pre-GE: T2 prepared gradient-echo; RF: Radio frequency; ROI: Region of interest; SI: Signal intensity; SNR: Signal-to-noise-ratio; STIR: Short tau inversion recovery; T2W: T2-weighted; TE: Echo time; TI: Inversion time; TR: Repetition time; TSE: Turbo spin echo; VENC: Velocity encoding value; VISTA: Volumetric isotropic turbo spin echo acquisition.

## Competing interests

The authors declare that they have no competing interests.

## Authors’ contributions

LØB carried out all the procedures related to the animal model, contributed to performing all the CMR scans, analyzed the acquired CMR data and histological data, contributed to the statistical analyses as well as elaboration of all figures and tables, wrote the manuscript, and merged all feedback from the co-authors into the final manuscript. AYKGH contributed to procedures related to the animal model, and drafting the manuscript and revised it critically for intellectual content. SFP contributed to the procedures related to the animal model and formulating the study design, and drafting the manuscript and revised it critically for intellectual content. JLH contributed to the study design, and drafting the manuscript and revised it critically for intellectual content. WYK took part in formulating the study design, the CMR sequence setup, drafting the manuscript and revised it critically and contributed to important intellectual content of the manuscript. ESSH contributed to the CMR sequence development and to performing the CMR scans as well as the analyses of the acquired CMR data and histological data, the statistical analyses, the figures and tables, drafting the manuscript and revised it critically for intellectual content. All authors have read and approved the final manuscript.
